# Supplementary material for: A multimodal sensing ring for quantification of scratch intensity
Source: Commun Med (Lond). 2023 Sep 19;3:115. doi: 10.1038/s43856-023-00345-2 (PMC10509275; doi:10.1038/s43856-023-00345-2)
Supplement: Supplementary file 1 — Reporting Summary [file 43856_2023_345_MOESM1_ESM.pdf]

nature portfolio

Corresponding author(s): Akhil Padmanabha

Last updated by author(s): Mar 5, 2023

Reporting Summary

Nature Portfolio wishes to improve the reproducibility of the work that we publish. This form provides structure for consistency and transparency in reporting. For further information on Nature Portfolio policies, see our Editorial Policies and the Editorial Policy Checklist.

Statistics

For all statistical analyses, confirm that the following items are present in the figure legend, table legend, main text, or Methods section.

n/a	Confirmed	
☐	☒	The exact sample size (*n*) for each experimental group/condition, given as a discrete number and unit of measurement
☒	☐	A statement on whether measurements were taken from distinct samples or whether the same sample was measured repeatedly
☐	☒	The statistical test(s) used AND whether they are one- or two-sided<br>*Only common tests should be described solely by name; describe more complex techniques in the Methods section.*
☒	☐	A description of all covariates tested
☒	☐	A description of any assumptions or corrections, such as tests of normality and adjustment for multiple comparisons
☐	☒	A full description of the statistical parameters including central tendency (e.g. means) or other basic estimates (e.g. regression coefficient) AND variation (e.g. standard deviation) or associated estimates of uncertainty (e.g. confidence intervals)
☒	☐	For null hypothesis testing, the test statistic (e.g. *F*, *t*, *r*) with confidence intervals, effect sizes, degrees of freedom and *P* value noted<br>*Give P values as exact values whenever suitable.*
☒	☐	For Bayesian analysis, information on the choice of priors and Markov chain Monte Carlo settings
☒	☐	For hierarchical and complex designs, identification of the appropriate level for tests and full reporting of outcomes
☐	☒	Estimates of effect sizes (e.g. Cohen's *d*, Pearson's *r*), indicating how they were calculated

Our web collection on statistics for biologists contains articles on many of the points above.

Software and code

Policy information about availability of computer code

Data collection	Python and various open-source libraries, Teensyduino. Code located here: https://github.com/RCHILab/Wearable Scratch Intensity
Data analysis	Python and various open-source libraries, Jupyter Notebook. Code located here: https://github.com/RCHILab/Wearable Scratch Intensity

For manuscripts utilizing custom algorithms or software that are central to the research but not yet described in published literature, software must be made available to editors and reviewers. We strongly encourage code deposition in a community repository (e.g. GitHub). See the Nature Portfolio guidelines for submitting code & software for further information.

Data

Policy information about availability of data

All manuscripts must include a data availability statement. This statement should provide the following information, where applicable:

- Accession codes, unique identifiers, or web links for publicly available datasets
- A description of any restrictions on data availability
- For clinical datasets or third party data, please ensure that the statement adheres to our policy

The datasets collected, generated, and analyzed during the current study are available on GitHub via this link: https://github.com/RCHILab/Wearable Scratch Intensity.

Human research participants

Policy information about studies involving human research participants and Sex and Gender in Research.

Reporting on sex and gender	In the manuscript, we have reported the gender of participants for both human studies conducted. We have not collected nor reported biological sex.
Population characteristics	In the manuscript, we have reported age and condition of participants.
Recruitment	Participants were recruited through word of mouth, email, and flyers.
Ethics oversight	Carnegie Mellon University Institutional Review Board

Note that full information on the approval of the study protocol must also be provided in the manuscript.

Field-specific reporting

Please select the one below that is the best fit for your research. If you are not sure, read the appropriate sections before making your selection.

☐ Life sciences ☒ Behavioural & social sciences ☐ Ecological, evolutionary & environmental sciences

For a reference copy of the document with all sections, see nature.com/documents/nr-reporting-summary-flat.pdf

Behavioural & social sciences study design

All studies must disclose on these points even when the disclosure is negative.

Study description	Both human studies were quantitative, experimental studies. For the first study, the participants were affixed with the wearable device and were instructed to do 7 scratching interactions and 7 non-scratching interactions. They were also instructed to scratch on a pressure sensitive tablet at combinations of force and velocity. For the second human study, the participants were affixed with the wearable device and instructed to scratch at intensities from 1-5 on a pressure sensitive tablet and on their skin at a location of their choosing.
Research sample	We recruited participants at Carnegie Mellon University. For our first human study (n=20), participant age was 21-30 (24.15 ± 2.52) and gender ratio (M:F) was 3:1. For the second human study (n=14), participant age was 18-32 (24.07 ± 4.57) and gender ratio (M:F:NB) was 9:4:1. The participant requirements were the following:<br>Participant must not have motor impairments.<br>and<br>Participant must be fluent in English.<br>and<br>Participants must be at least 18 years old<br>and<br>Participant must not have cognitive impairments<br>and<br>Participant must not have visual impairments (impairments that are not correctable by conventional means).<br>and<br>Participant must never have had a diagnosis of eczema, psoriasis, kidney/liver disease, or any medical condition that may cause dry, itchy skin or skin condition that may be inflamed by scratching or rubbing.<br>and<br>Participant must not have had any itchy skin or rash in the past 6 months.<br>and<br>Participant must not have seen a dermatologist for a skin condition in the last 3 years.
Sampling strategy	A sample size of 20 participants was used for our first human study to allow for robust evaluation of our algorithms using leave one subject out cross validation. A sample size of 14 participants for the second human study was sufficient data for validation of our scratch intensity algorithm using the Wilcoxon signed rank test and Pearson's correlation (140 points).
Data collection	Data was collected using a custom wearable device detailed in the manuscript. The ring was fabricated using the Sparkfun ADXL362 3-axis accelerometer breakout board, PUI Audio, Inc AB1070B-LW100-R contact microphone, Teensy 4.0 microcontroller, and a custom printed circuit board. A python script was used to collect and save data.
Timing	first study (03/2022-05/2022), second study (10/2022-12/2022)
Data exclusions	Three participants' data was disregarded from the first human study due to sensor failures.
Non-participation	One participant was dis-enrolled from the first human study prior to data collection for not meeting inclusion criteria.

Randomization	Subjects were not allocated into experimental groups.

Reporting for specific materials, systems and methods

We require information from authors about some types of materials, experimental systems and methods used in many studies. Here, indicate whether each material, system or method listed is relevant to your study. If you are not sure if a list item applies to your research, read the appropriate section before selecting a response.

Materials & experimental systems

n/a	Involved in the study
☒	☐ Antibodies
☒	☐ Eukaryotic cell lines
☒	☐ Palaeontology and archaeology
☒	☐ Animals and other organisms
☒	☐ Clinical data
☒	☐ Dual use research of concern

Methods

n/a	Involved in the study
☒	☐ ChIP-seq
☒	☐ Flow cytometry
☒	☐ MRI-based neuroimaging
