# Supplementary material for: A multimodal sensing ring for quantification of scratch intensity
Source: Commun Med (Lond). 2023 Sep 19;3:115. doi: 10.1038/s43856-023-00345-2 (PMC10509275; doi:10.1038/s43856-023-00345-2)
Supplement: Supplementary file 12 — Supplementary Information [file 43856_2023_345_MOESM12_ESM.pdf]

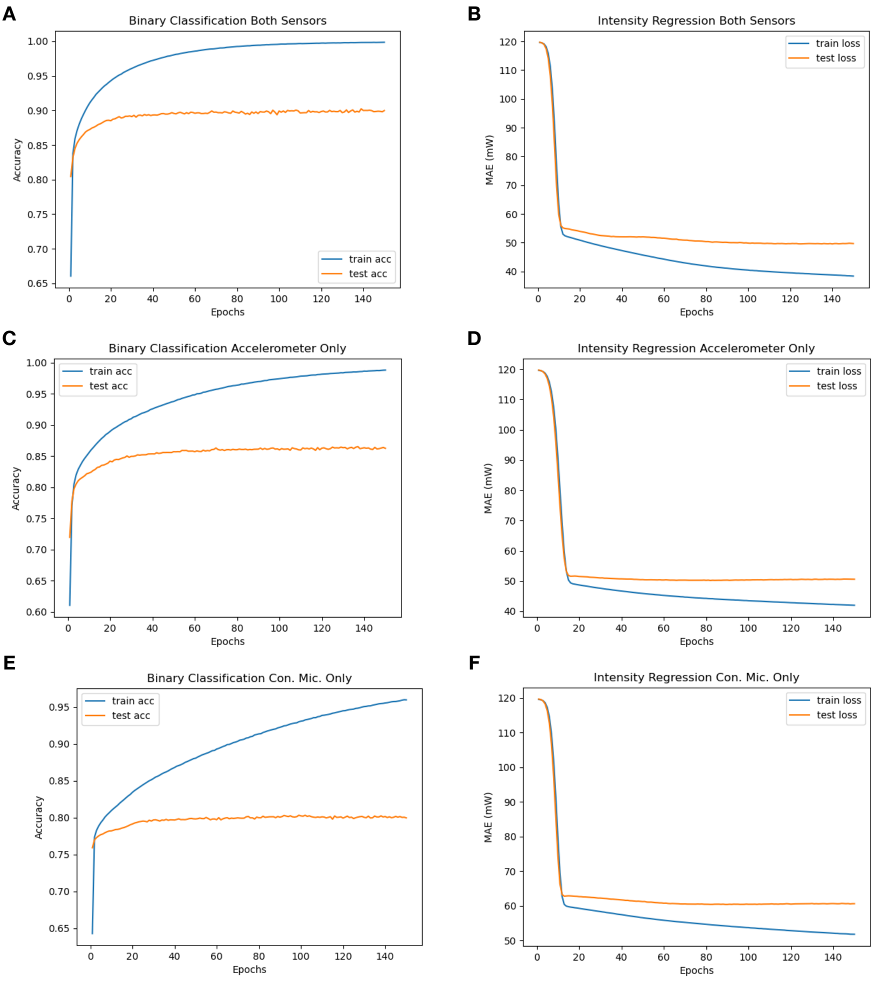

Fig. S1 Train/Test Plots. These train-test plots were generated during training for the 3 scratch intensity models and the 3 scratch detection models. The train and test curves for each plot are averaged across all 20 folds. **A.** Scratch Detection Train-Test Plot for Acc+Con Model. **B.** Intensity Regression Train-Test Plot for Acc+Con Model. **C.** Scratch Detection Train-Test Plot for Acc Only Model. **D.** Intensity Regression Train-Test Plot for Acc Only Model. **E.** Scratch Detection Train-Test Plot for Con Only Model. **F.** Intensity Regression Train-Test Plot for Con Only Model.

Table S1 Intensity Regression: LOSO-CV Results per Participant from Study 1. Detailed results for all intensity regression models for all 20 participants from study 1. The min, max, mean, and standard deviation are all shown as individual rows. The results for each participant were generated using LOSO-CV. For each fold, a participant was held-out as a test set while the model was trained on the other 19 participants.

Participant	Acc+Con MAE(mW)	Acc+Con MAPE(%)	Acc MAE(mW)	Acc MAPE(%)	Con MAE(mW)	Con MAPE(%)	Naive Predictor MAE(mW)	Naive Predictor MAPE(%)
P1	51	8.5	49.53	8.26	78.88	13.15	88.25	14.71
P2	81.28	13.55	74.05	12.34	90.12	15.02	67.48	11.25
P3	50.44	8.41	35.96	5.99	90.85	15.14	79	13.17
P4	49.07	8.18	53.34	8.89	28	4.67	75.26	12.54
P5	37.08	6.18	39.91	6.65	44.57	7.43	91.46	15.24
P6	76.45	12.74	86.55	14.42	54.71	9.12	75.5	12.58
P7	14.01	2.33	13.9	2.32	27.93	4.65	56.67	9.44
P8	33.32	5.55	31.38	5.23	49.95	8.32	85.74	14.29
P9	45.22	7.54	56.48	9.41	81.51	13.59	75.02	12.5
P10	36.69	6.12	52.26	8.71	54.51	9.08	96.18	16.03
P11	42.09	7.01	45.1	7.52	54.24	9.04	86.77	14.46
P12	85.73	14.29	81.74	13.62	72.59	12.1	115.13	19.19
P13	60.87	10.15	56.12	9.35	45.61	7.6	60.62	10.1
P14	86.65	14.44	94.79	15.8	112.2	18.7	144.69	24.12
P15	26.59	4.43	29.8	4.97	72.37	12.06	54.58	9.1
P16	19.57	3.26	27.1	4.52	33.27	5.54	109.25	18.21
P17	19.59	3.26	21.02	3.5	32.72	5.45	56.32	9.39
P18	29.45	4.91	30.96	5.16	37.01	6.17	61.69	10.28
P19	45.95	7.66	41.19	6.86	73.72	12.29	70.76	11.79
P20	103.21	17.2	90.53	15.09	78.37	13.06	166.43	27.74
**Min**	14.01	2.33	13.9	2.32	27.93	4.65	54.58	9.1
**Max**	103.21	17.2	94.79	15.8	112.2	18.7	166.43	27.74
**Mean**	49.71	8.29	50.58	8.43	60.66	10.11	85.84	14.31
**SD**	24.58	4.1	23.29	3.88	23.35	3.89	28.61	4.77

Table S2 Intensity Regression: Validation Results per Participant from Study 2. Detailed results for intensity regression for all 14 participants from study 2. Data from these participants was used to validate the Acc+Con model trained with data from all 20 participants from study 1. The min, max, mean, and standard deviation are all shown as individual rows.

Participant	Acc+Con MAE(mW)	Acc+Con MAPE(%)	Naive Predictor MAE(mW)	Naive Predictor MAPE(%)
P1	43.25	7.21	86.76	14.46
P2	17.5	2.92	76.29	12.71
P3	69.07	11.51	139.54	23.26
P4	28.21	4.7	58.69	9.78
P5	144.88	24.15	68.62	11.44
P6	39.87	6.64	80.95	13.49
P7	69.81	11.63	40.83	6.8
P8	29.57	4.93	70.83	11.81
P9	111.84	18.64	57.39	9.57
P10	55.04	9.17	125.15	20.86
P11	49.29	8.21	69.84	11.64
P12	59.13	9.85	104.04	17.34
P13	35.35	5.89	110.8	18.47
P14	50.77	8.46	92.73	15.45
**Min**	17.5	2.92	40.83	6.8
**Max**	144.88	24.15	139.54	23.26
**Mean**	57.4	9.57	84.46	14.08
**SD**	32.99	5.5	26.55	4.43

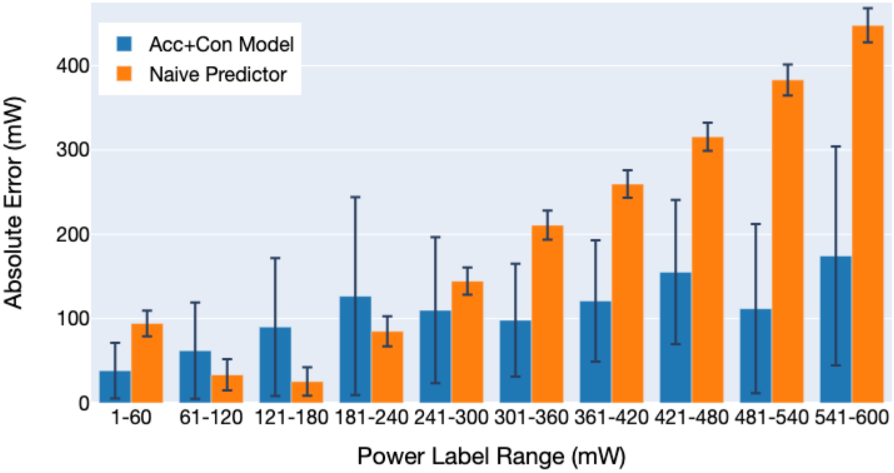

Fig. S2 Validation Error for Ranges of Power Labels. Test error in predictions as power labels increase for scratch intensity data from the 14 participants from our second study. Error bars indicate 1 standard deviation from the mean. Our method performs better than the naive predictor for every range except for the $61 - 120$, $121 - 180$, and $181 - 240$ mW ranges. In comparison to the LOSO-CV errors, shown in Fig. 4H in the main text, we notice higher errors in the lower power ranges and lower errors in the higher power ranges. Underlying data for this figure can be found in Supplementary Data 10.

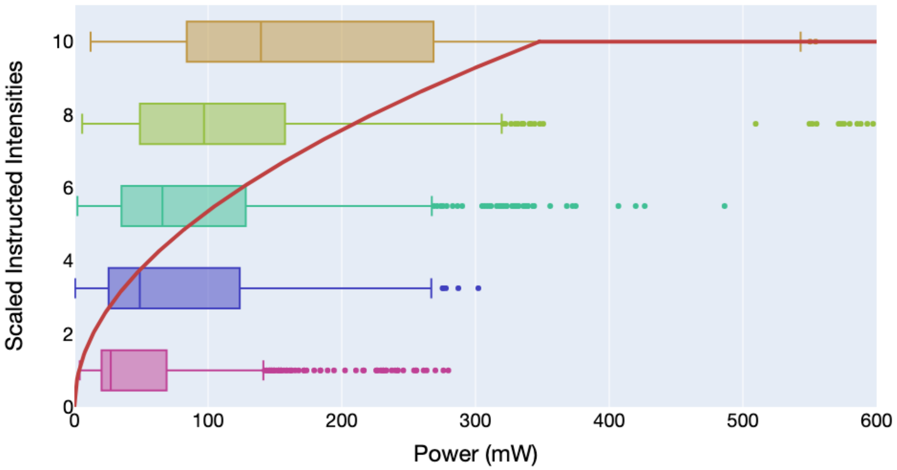

Fig. S3 Non-Linear Function for Conversion of Power Scale to 0-10 Scale. We can apply mapping functions other than the linear function presented in the main text to convert from the 0-600 mW scale to the 0-10 continuous scale. As shown, an alternative is to fit a square root function of form $y = a\sqrt{x}$ to ground truth power labels from the 2 sets of scratching on the pressure sensitive tablet by the 14 participants. The fitted function is $y = 0.536\sqrt{x}$. The bounds in power units for the 0-10 scale are the following: 0, 3.48, 13.91, 31.30, 55.64, 86.94, 125.20, 170.40, 222.57, 281.69, and 347.77 mW. Because the 0-10 continuous scale ends at 10, anything over 347.77 mW is saturated at 10. Using the fitted function, we convert the ground truth power labels in the 0-600 mW power scale to the 0-10 continuous scale; and we observe a MAE of 1.37 units with a standard deviation of 0.56.

Table S3 Scratch Detection: LOSO-CV Results per Participant. Detailed results for all scratch detection models for all 20 participants from study 1. The min, max, mean, and standard deviation are all shown as individual rows. The results for each participant were generated using LOSO-CV. For each fold, a participant was held-out as a test set while the model was trained on the other 19 participants. Note: the participant numbers here do not match the participant numbers in Fig. 5E.

Participant	Acc+Con Accuracy(%)	Acc Accuracy(%)	Con Accuracy(%)
P1	94.14	86.51	76.43
P2	88.52	87.79	77.96
P3	82.6	75.34	79.67
P4	83.09	84.68	79.91
P5	94.38	87.85	79.37
P6	93.35	80.95	77.78
P7	98.05	86.14	86.75
P8	94.81	83.03	84.19
P9	90.78	86.26	91.88
P10	84.74	85.1	72.1
P11	95.48	93.89	85.59
P12	94.51	93.77	86.08
P13	94.14	89.44	86.69
P14	96.28	91.58	83.27
P15	80.22	82.6	71.18
P16	93.47	91.88	79.49
P17	81.14	84.98	69.78
P18	91.58	85.96	83.27
P19	84.74	88.03	74.05
P20	83.58	79.06	74.05
**Min**	80.22	75.34	69.78
**Max**	98.05	93.89	91.88
**Mean**	89.98	86.24	79.98
**SD**	5.63	4.59	5.82
