# Supplementary material for: A multimodal sensing ring for quantification of scratch intensity
Source: Commun Med (Lond). 2023 Sep 19;3:115. doi: 10.1038/s43856-023-00345-2 (PMC10509275; doi:10.1038/s43856-023-00345-2)
Supplement: Supplementary file 14 — Description of Additional Supplementary Files [file 43856_2023_345_MOESM14_ESM.pdf]

Description of Additional Supplementary Files

File name: Supplementary Movie
Description: The top of the video shows a subject demonstrating scratching behavior with only finger movement and scratching behavior with only arm movement. The footage of the subject is synchronized with contact microphone and accelerometer (z-axis) raw data signals which are visualized in the bottom half of the video. From 0-1 seconds, the subject moves their hand towards their arm to begin the scratching motion. From 1-6 seconds, the subject scratches with only finger movement. From 6-13 seconds, the subject pauses scratching. From 13-20 seconds, the subject scratches with only arm movement.

File name: Supplementary Data 1
Description: Underlying data for Figure 4d.

File name: Supplementary Data 2
Description: Underlying data for Figure 4e.

File name: Supplementary Data 3
Description: Underlying data for Figure 4f.

File name: Supplementary Data 4
Description: Underlying data for Figure 4h.

File name: Supplementary Data 5
Description: Underlying data for Figure 5c.

File name: Supplementary Data 6
Description: Underlying data for Figure 5d.

File name: Supplementary Data 7
Description: Underlying data for Figure 5e.

File name: Supplementary Data 8
Description: Underlying data for Figure 5f.

File name: Supplementary Data 9
Description: Underlying data for Figure 6e.

File name: Supplementary Data 10
Description: Underlying data for Figure S2.
